# Transcriptome analyses of adult mouse brain reveal enrichment of lncRNAs in specific brain regions and neuronal populations

**DOI:** 10.3389/fncel.2015.00063

**Published:** 2015-03-06

**Authors:** Beena M. Kadakkuzha, Xin-An Liu, Jennifer McCrate, Gautam Shankar, Valerio Rizzo, Alina Afinogenova, Brandon Young, Mohammad Fallahi, Anthony C. Carvalloza, Bindu Raveendra, Sathyanarayanan V. Puthanveettil

**Affiliations:** ^1^Department of Neuroscience, The Scripps Research InstituteJupiter, FL, USA; ^2^Informatics Core, The Scripps Research InstituteJupiter, FL, USA; ^3^Genomics Core, The Scripps Research InstituteJupiter, FL, USA

**Keywords:** lncRNA, mRNA, genomic context, hippocampus, pre-frontal cortex, differential gene expression, RNAseq, pathway analysis

## Abstract

Despite the importance of the long non-coding RNAs (lncRNAs) in regulating biological functions, the expression profiles of lncRNAs in the sub-regions of the mammalian brain and neuronal populations remain largely uncharacterized. By analyzing RNASeq datasets, we demonstrate region specific enrichment of populations of lncRNAs and mRNAs in the mouse hippocampus and pre-frontal cortex (PFC), the two major regions of the brain involved in memory storage and neuropsychiatric disorders. We identified 2759 lncRNAs and 17,859 mRNAs in the hippocampus and 2561 lncRNAs and 17,464 mRNAs expressed in the PFC. The lncRNAs identified correspond to ~14% of the transcriptome of the hippocampus and PFC and ~70% of the lncRNAs annotated in the mouse genome (NCBIM37) and are localized along the chromosomes as varying numbers of clusters. Importantly, we also found that a few of the tested lncRNA-mRNA pairs that share a genomic locus display specific co-expression in a region-specific manner. Furthermore, we find that sub-regions of the brain and specific neuronal populations have characteristic lncRNA expression signatures. These results reveal an unexpected complexity of the lncRNA expression in the mouse brain.

## Introduction

While previously regarded as simply “transcriptional noise,” non-coding RNAs (ncRNAs) constitute 97–98% of the human genome that is not translated into proteins (Derrien et al., 2012). Long non-coding RNAs (lncRNAs) are transcribed by RNA polymerases II and III (Dieci et al., 2007; Kapranov et al., 2007; Guttman et al., 2009), and are a minimum of 200 nucleotides long. Closer scrutiny of lncRNAs, spurred by recent technological advances in DNA microarrays and RNA sequencing, has suggested that they are involved in chromatin remodeling, transcription, alternative splicing, imprinting, cytoplasmic trafficking, and aging (Mercer et al., 2009; Wang and Chang, 2011; Soreq et al., 2014). Based on the genomic orientation of lncRNAs and their associated protein-coding genes, lncRNAs are characterized as intronic, bidirectional, antisense, sense overlapping, and intergenic (Ma et al., 2013).

Whole transcriptome sequencing to investigate tissue-specific expression in lower organisms found that the largest number of tissue specific lncRNA transcripts is expressed in the brain (Kaushik et al., 2013). The large number of lncRNAs that exhibit neuronal-specific expression suggests their essential role in the neuronal diversification seen in higher vertebrates (Cao et al., 2006; Qureshi et al., 2010). It has been reported that aberrant expression of lncRNAs is associated with neurological disorders (Faghihi et al., 2008; Khalil et al., 2009; Johnson, 2012; Pauli et al., 2012; Roberts et al., 2014; Soreq et al., 2014). Using the *in situ* hybridization data available from the Allen Brain Atlas, Mercer et al., reported that lncRNAs are differentially expressed in different regions of the adult mouse brain (Mercer et al., 2008). Recently, Sauvageau et al., have developed several lines of knockout mice to investigate a family of non-coding RNA molecules known as long intergenic non-coding RNAs (lincRNAs) (Sauvageau et al., 2013). These studies reveal that lincRNAs regulate key functions of the overall viability and developmental processes of mice, including the development of the cerebral cortex.

While functional characterization of neuronal-enriched lncRNAs is still limited, broader studies of lncRNA function have implicated them as regulators of transcription through epigenetic regulation of chromatin structure and RNA-transcription factor interaction (Kurokawa, 2011; Kornienko et al., 2013; Wu et al., 2013; Halley et al., 2014). Despite this importance we still do not have unbiased estimates of lncRNAs expressed in specific brain regions and the extent of their differential expression in mammalian brain.

Here we report the identification of the lncRNAs and mRNAs expressed in two key brain regions involved in memory and neuropsychiatric disorders, the hippocampus and pre-frontal cortex (PFC). Following RNAseq analysis (*N* = 4 biological replicates) of hippocampus and PFC, we identified 2759 lncRNAs expressed in the hippocampus and 2561 lncRNAs in PFC using a customized computational analysis pipeline and have identified their chromosomal locations. Among these, 2390 lncRNAs are expressed in both regions. Importantly, 24 lncRNA transcripts are differentially expressed in the hippocampus and PFC. We have then studied differential expression of a subset of lncRNAs in (1) two other brain regions, striatum and cerebellum, (2) dorsal and ventral hippocampus, (3) three different neuronal populations (CA1, CA3, and dentate gyrus neurons of hippocampus), and (4) have examined subcellular distribution of a subset of candidates in the hippocampus and PFC by RNA *in situ* hybridization and sub-cellular fractionation. Our studies establish that specific brain regions and neuronal populations have characteristic expression pattern of lncRNAs.

## Results

### Overview of lncRNA and mRNA profiles in adult mouse hippocampus and PFC

**Figure 1:**
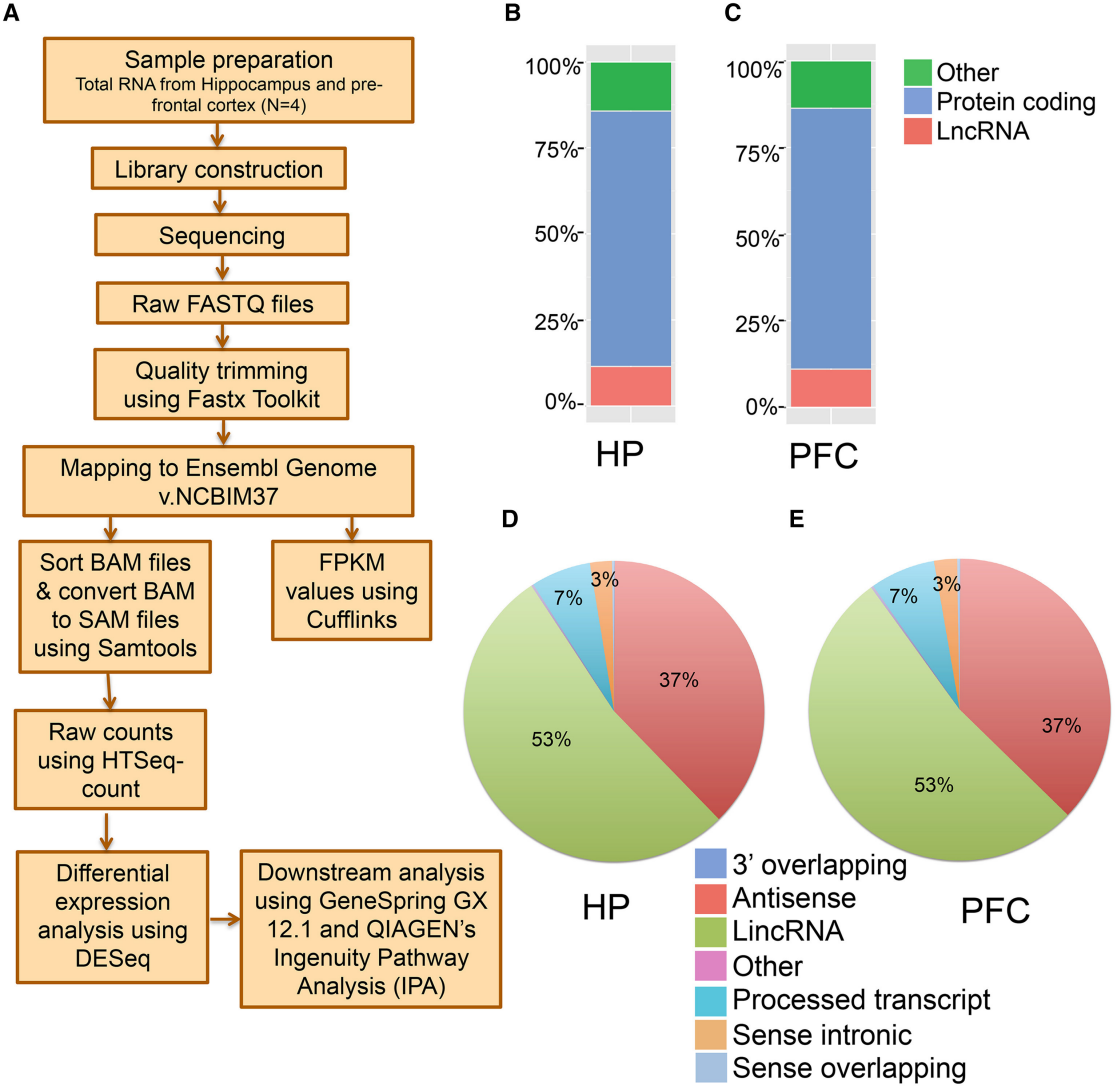
**Next generation sequencing analysis of hippocampus (HP) and pre-frontal cortex (PFC) from adult mouse brain**. **(A)** Pipeline for the identification of mRNAs and lncRNAs from mouse brain total RNA preparation. Total RNA was isolated from HP and PFC for RNAseq (*N* = 4). **(B, C)** Classification of whole transcriptome from HP and PFC, respectively, based on the coding potential of the transcripts mapped. **(D, E)** Pie charts showing the percentage of seven categories of lncRNAs identified in HP and PFC on the basis of their relationship with protein coding genes.

To perform an unbiased profiling of the total transcriptome of the hippocampus and pre-frontal cortex (PFC) in the adult mouse, RNA samples were prepared from the respective brain regions of 7–9 week old male mice (*N* = 4 biological replicates, each replication has two mice). The transcripts were assembled and analyzed using a reported workflow as shown in Figure 1A. We mapped a total of 20,618 genes in the hippocampus and 20,025 genes in the PFC, including both protein-coding and non-coding transcripts (Table S1). There are 3823 lncRNAs so far annotated in the mouse genome, according to the NCBIM37, and the 2759 lncRNAs mapped in the hippocampus and 2561 in the PFC from our study represent ~70% of the total annotated mouse lncRNAs. Alignment of mapped reads from each sample to mouse transcriptome is provided in Table S1. Additionally, these lncRNAs represent ~14% of the total mouse brain transcriptome mapped in this study. The total transcriptome composition in each tissue is shown in Figures 1B,C for the hippocampus and PFC, respectively. Next, we estimated the expression abundance of all lncRNA and mRNA genes by running Cufflinks across 8 samples using the ENSEMBL annotation file (NCBI37).

Analysis of the top 50 lncRNAs based on expression abundance identified key members such as *MALAT1, MEG3, RIAN, XIST, MIAT, and TUG1* (Figures S1A,B). *MALAT1* knockout mice studies showed that *MALAT1* neighboring genes were deregulated, indicating a potential cis-regulatory role of *MALAT1* in gene transcription (Zhang et al., 2012). The Allen Brain Atlas shows a similar expression profile for the lncRNAs *MEG3, RIAN*, and *MIAT*. Additionally, we looked at the top 50 mRNAs and the complete list is represented in Figures S1C,D. This abundance estimation indicated much lower expression levels of non-coding RNAs compared to that of protein-coding RNAs and that most lncRNAs (>80%) are expressed at a low level, an observation similar to those drawn from previous studies of the mammalian genome (Mercer et al., 2008; Derrien et al., 2012).

**Figure 2:**
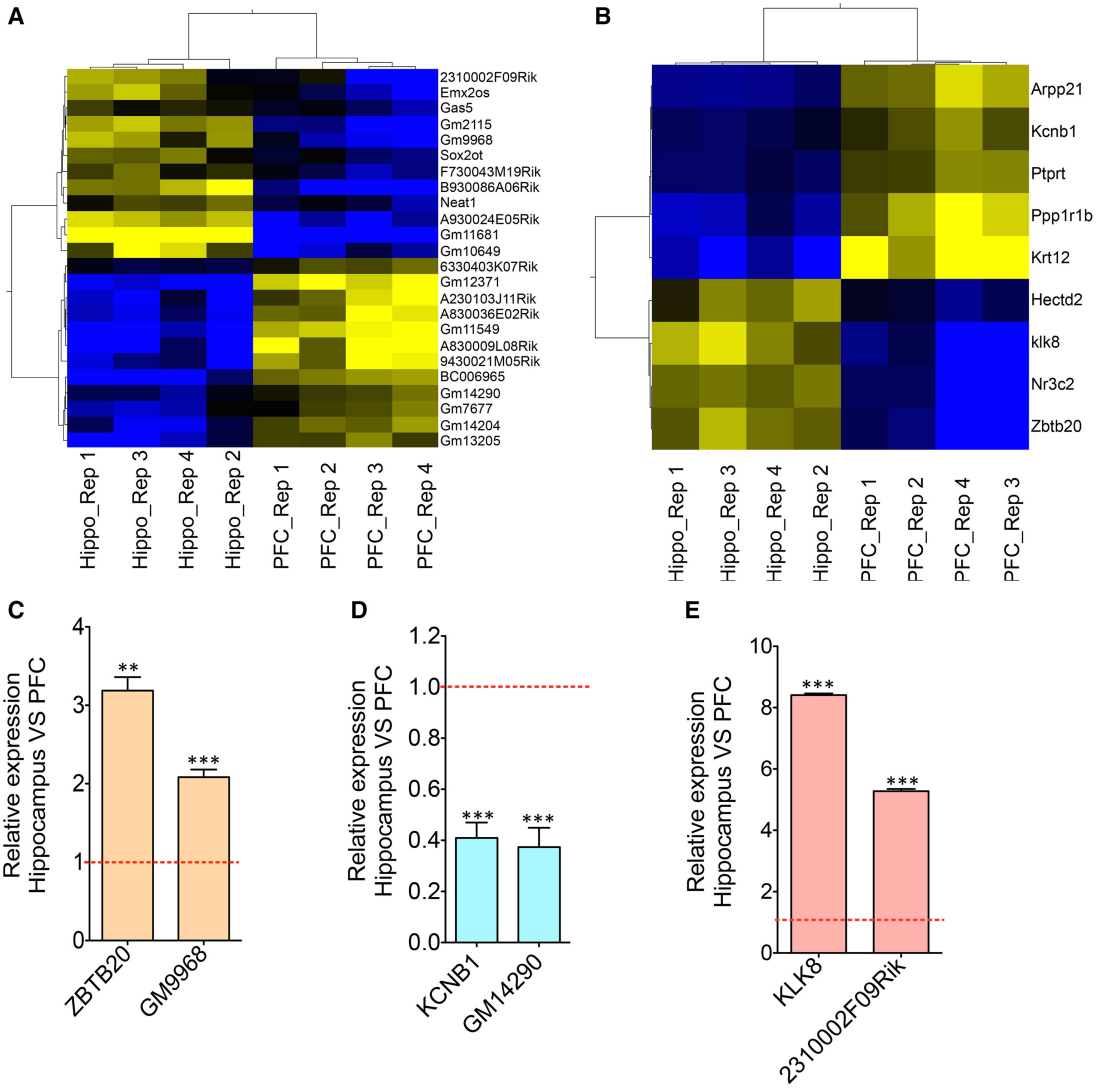
**Co-expression analyses of differentially expressed lncRNAs and neighboring mRNAs. (A)** Heat map showing differentially expressed lncRNAs between HP and PFC (*N* = 4). **(B)** Heat map showing mRNAs that are differentially expressed between HP and PFC as well as that present within a 50 Kb genomic region flanking the genomic locus of differentially expressed lncRNAs in **(A)**. **(C–E)** Are the qRT-PCR validation of the expression pattern of three lncRNA-mRNA pairs (GM9968 and ZBTB20; GM14290 and KCNB1; 2310002F09Rik and KL8) showing concordant gene expression between the lncRNA and its overlapping mRNA (*N* = 4; Student *t*-test, ^**^P-value < 0.01, ^***^P-value < 0.001). Data was normalized to 18sRNA level. Red dotted line shows 1-fold change. Error bars represent SEM.

The transcriptomic profile of mRNA and lncRNA expression is summarized in Table S1. Further classification of the lncRNAs based on genomic context using the ENSEMBL annotation file provided eight biotypes: antisense, long lincRNAs, processed transcripts, sense overlapping, sense intronic, long non-coding, 3′ overlapping ncRNA and ncRNA host. Among these, lincRNAs (53%) and antisense ncRNAs (38%) showed predominance compared to other types of ncRNAs in both tissues (Figures 1D,E), an outcome similar to the observations from earlier studies (Khalil et al., 2009; Pauli et al., 2012; Ma et al., 2013). For example, the human GENCODE catalog mapped more than 70% of the lncRNAs into the lincRNA category (Derrien et al., 2012). Similarly the transcriptomic profiling in Maize showed that 93% of lncRNAs are located in intergenic regions and only 7% of the lncRNAs overlap with gene sequences (Li et al., 2014). To estimate the differential expression (DE), the raw read counts generated in HTSeq were used in the R Bioconductor package DESeq. DESeq analyses of the protein coding as well as non-coding transcripts showed a robust DE pattern where 24 long non-coding (Figure 2A) and 821 protein-coding transcripts were differentially expressed between the hippocampus and PFC (adjusted *p*-value < 0.05; Table S1).

### Chromosome organization of lncRNA genes in mouse brain

**Figure 3:**
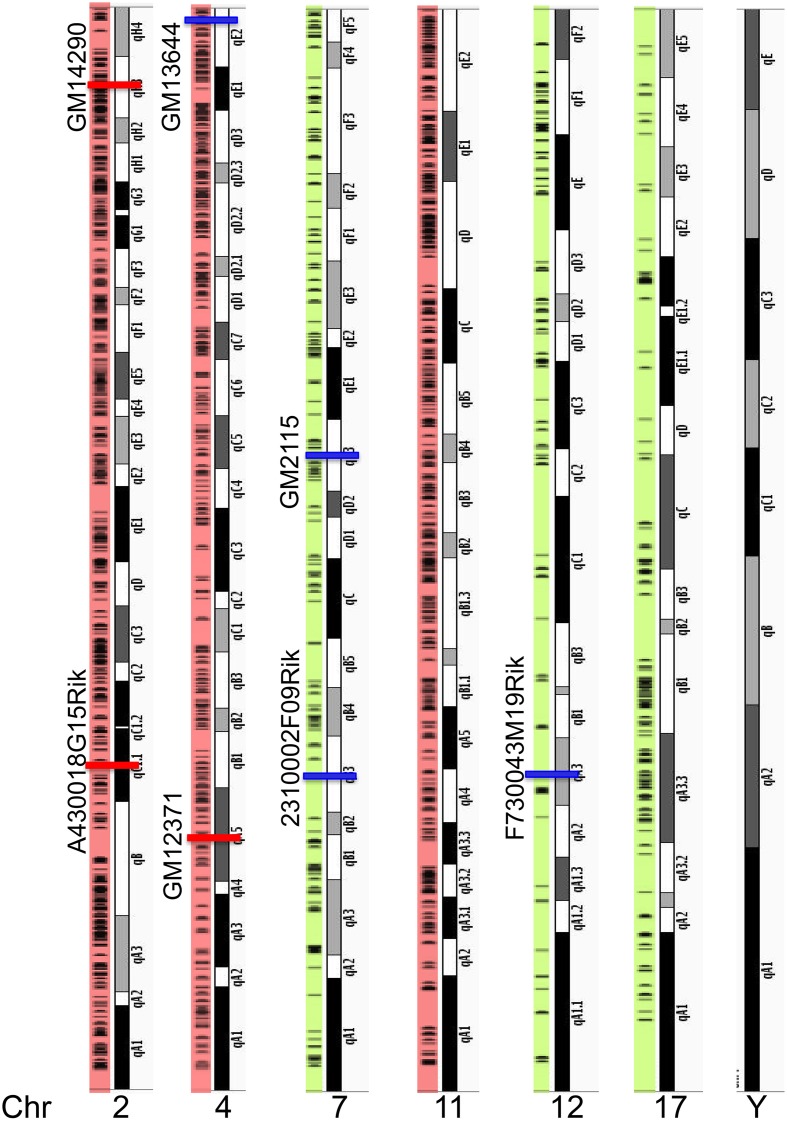
**Chromosome locations of all lncRNAs along mouse genome**. Top three chromosomes (2, 4, and 11) that encode maximum number of lncRNAs are highlighted in red and three examples of chromosomes that code less than 150 lncRNAs are highlighted in green. Horizontal black lines in the color shaded along the chromosome show chromosomal location of individual lncRNAs. This analysis showed clustering of several lncRNAs along the chromosomes. All the 21 chromosomes and their lncRNAs distribution are illustrated in Figure S3. Percentage of lncRNAs in each chromosome is provided in Table S2. Chromosomal locations of seven lncRNAs used in this study and their genomic location (Red: enriched in PFC and blue: enriched in HP) are indicated in the Figure. None of the lncRNAs we identified in the HP and PFC map onto the Y chromosome.

Next, we asked whether the differentially enriched lncRNAs in the hippocampus and PFC are clustered in a specific region or randomly distributed on chromosomes. In order to observe the chromosomal distribution of lncRNAs, we mapped the transcriptional loci of all 2930 lncRNAs detected in this study. Table S2 list the number of lncRNAs expressed from each chromosome from the current study and Figure S3 shows the distribution of lncRNAs on each chromosome. Based on the number of lncRNAs encoded, the top three chromosomes (2, 4, and 11) are highlighted in red. A “spot” on the chromosome where we observe a large number of lncRNAs are clustered together, we termed as an “lncRNA transcriptional cluster” while discussing the chromosomal distribution of lncRNAs in the manuscript. Figure 3 shows that most of the lncRNA loci mapped in this study are clustered along chromosomes 2, 4, and 11. LncRNA loci in other chromosomes are shown in Figure S3. We find that though lncRNA loci are present as several clusters, the genomic loci of differentially expressed lncRNAs in the hippocampus and PFC are randomly distributed along the chromosomes (red: higher in PFC and blue: higher in hippocampus). Interestingly, we did not find any hippocampal or PFC lncRNAs present in the Y chromosome. Similar to our finding, an earlier study on the mouse embryonic brain also did not assign any lncRNAs to the Y chromosome (Lv et al., 2013).

### Expression analysis of differentially enriched protein-coding-noncoding genes that share common genomic locus

We analyzed whether the differentially enriched lncRNAs and the mRNAs within its vicinity show co-enrichment in these brain regions. Selective enrichment of lncRNA and neighboring mRNAs suggest co-regulation of their expression. We determined which of the 24 differentially expressed lncRNAs are transcribed from genomic loci overlapping with, or adjacent to, the loci that encode 821 differentially expressed mRNAs identified from DESeq analysis. For the purpose of defining “genomic association,” we have defined 50 Kb in either direction of the genomic loci of a given lncRNA (Figure 2B) as the “neighboring chromosomal region.” Furthermore, we considered that the DE of one of the genes in this pair (lncRNA and mRNA) is either positively or inversely correlated with the DE of the other gene.

By screening ~50 Kb genomic region flanking the genomic loci of all 24 lncRNAs on either direction using the UCSC genome database, (https://genome.ucsc.edu/), we identified ~50 mRNA genes. Matching these ~50 mRNAs to the differentially enriched 821 mRNA gene list identified 9 mRNAs, that are within 50 kb of lncRNAs and are differentially enriched between the hippocampus and PFC. For further characterization we have selected from this list, lncRNAs and their corresponding mRNAs coded from the same loci. We found three lncRNA-mRNA pairs that share a common genomic locus and are partially or completely overlapping with each other.

One of these three pairs, *GM9968*, is an lncRNA significantly enriched in the mouse hippocampus and transcribed in the antisense direction of DNA binding protein gene *ZBTB20* (Zinc finger and BTB domain-containing protein 20). Past studies showed that *ZBTB20* is particularly expressed in primary hippocampal neurons, cerebellar granule cells, gliogenic progenitors and differentiated glia. RNAseq analyses showed that both of these transcripts are enriched in the hippocampus, and we confirmed the results by qRT-PCR, which showed a 2-fold enrichment of *GM9968* and 3-fold enrichment of *ZBTB20* in the hippocampus compared to the PFC (Figure 2C). In a similar way we analyzed two more protein-coding lncRNA pairs to identify specific expression patterns. *GM14290* is another lncRNA on chromosome 2 that shares exonic overlap with the gene *KCNB1. KCNB1* is a potassium voltage-gated channel subfamily B member 1 that mediates the voltage-dependent potassium ion permeability of excitable membranes. Interestingly, the *GM14290*-*KCNB1* pair is enriched in the PFC compared to the hippocampus and we have confirmed the NGS results with qRT-PCR (Figure 2D). Additionally, our studies showed that *2310002F09Rik* is another lncRNA that shares an exonic overlap with *KLK8* in the antisense direction. *KLK8* (kallikrein-related peptidase 8), known commonly as neuropsin, is a serine protease expressed predominantly in the brain. Earlier analyses using northern blot and *in situ* hybridization demonstrated that neuropsin is expressed specifically in the limbic system of the mouse brain and is present at the highest concentrations in pyramidal neurons of the hippocampal CA1 and CA3 subfields (Oka et al., 2002).

Consistent with the above studies, our gene expression analyses, both by RNAseq as well as qRT-PCR validated that klk8/neuropsin is highly enriched in the hippocampus (~5-fold difference) compared to the PFC. We found that the lincRNA *2310002F09Rik* that shares exonic overlap with neuropsin spans the promoter region of the neuropsin and is enriched in the hippocampus (~8-fold) compared to the PFC. Both genes are enriched very significantly in the hippocampus compared to the PFC. Here, these three pairs had a positive, direct correlation where both mRNA and lncRNA were selectively enriched in either the hippocampus (*GM9968* -*ZBTB20 and 2310002F09Rik* -*KLK8)* or PFC (*GM14290*-*KCNB1)*. This observation is consistent with the prevalence of concordant expression patterns in mammalian *cis*-sense or *cis*-antisense gene pairs (Nakagawa and Kageyama, 2014).

To understand the functional significance of the tissue-specific gene signatures, we applied gene ontology enrichment analysis of upregulated (log2 fold change > 1, adjusted *p*-value < 0.05) and downregulated genes (log2 fold change < –1, adjusted *p*-value < 0.05) in the hippocampus compared to the PFC using Ingenuity Pathway Analysis (Qiagen-IPA). The top ten upregulated and downregulated pathways were identified and sorted according to their statistical significance as the negative logarithm of *p*-values calculated by IPA (Figures S4A,B). Our analysis found that many of the differentially expressed mRNAs assigned to the canonical pathway mapping are involved in the categories “Neurological diseases, Psychological diseases and Behavior” and are enriched in the PFC compared to the hippocampus.

### Dynamic expression pattern of lncRNAs in different mouse brain regions

**Figure 4:**
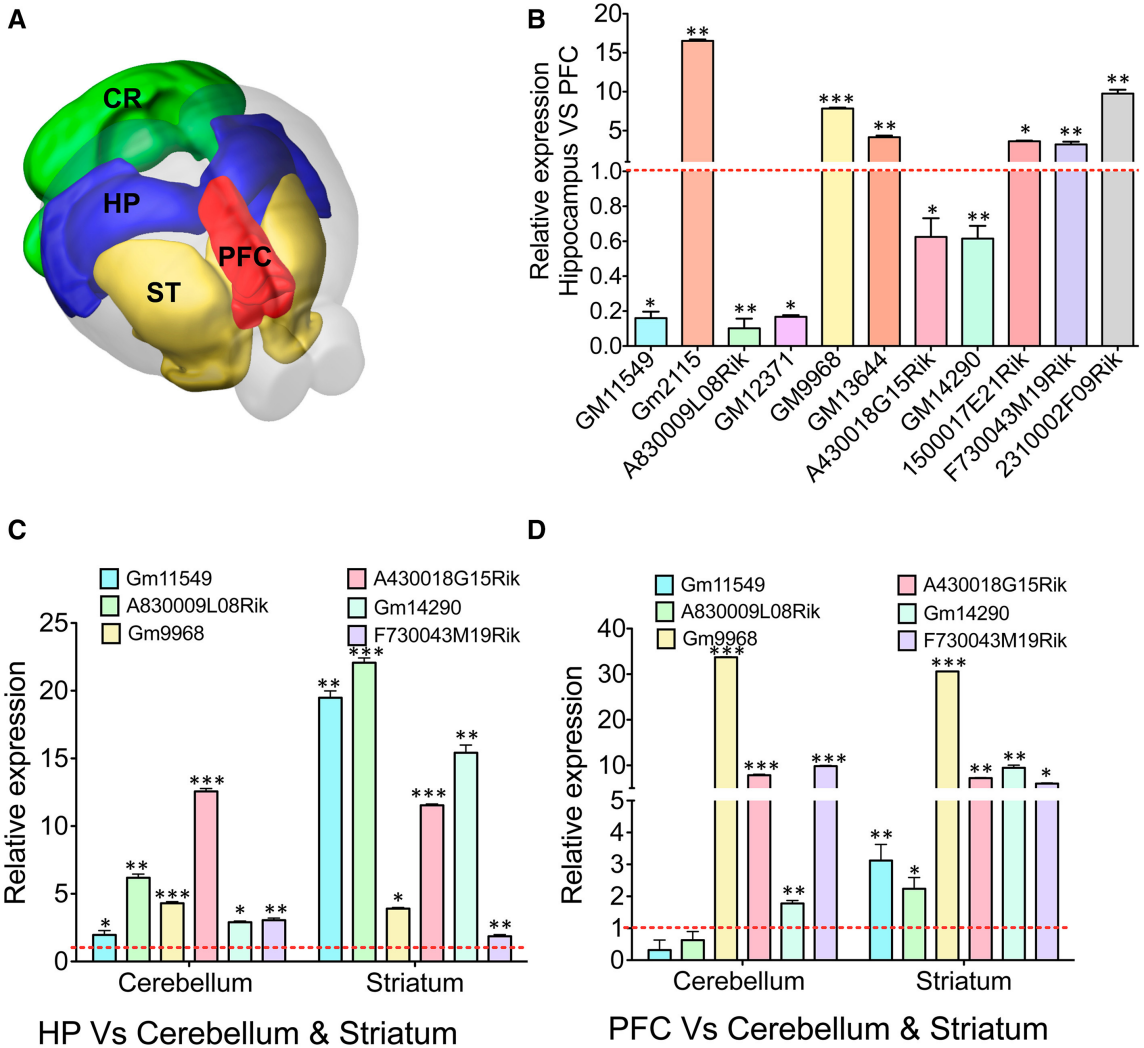
**qRT-PCR validation of differentially expressed lncRNAs in different brain regions. (A)** Cartoon showing different subregions (ST: striatum, PFC: pre-frontal cortex, HP: hippocampus CR: cerebellum) of the adult mouse brain that was used to study expression of several lncRNAs identified from RNAseq analysis of the HP and PFC. **(B)** Bar graphs showing the qRT-PCR validation of 11 lncRNAs between the hippocampus and PFC from RNAseq analysis (adjusted *p*-value < 0.05). From the list of 11 lncRNAs, we selected six lncRNAs to investigate their expression profile in regions in striatum and cerebellum. **(C, D)** Show the expression pattern of six lncRNAs in cerebellum and striatum compared to hippocampus and PFC, respectively (*N* = 4; Student *t*-test; ^*^P-value < 0.05, ^**^P-value < 0.01, ^***^P-value < 0.001). Data was normalized to 18sRNA level. Red dotted line corresponds to 1-fold change. Error bars represent SEM.

We next studied whether the selectively enriched lncRNAs in the hippocampus and PFC show DE in other regions of the brain (Figure 4A). We have selected 11 lncRNA genes, six of them enriched in the hippocampus and five enriched in the PFC as outlined in Figure 4B and studied their expression in striatum and cerebellum. LncRNAs with a very low expression based on FPKM values were excluded to avoid stochastic biases generated during qRT-PCR amplification. qRT-PCR analyses of these samples were then compared to the RNAseq data for both tissue types.

To corroborate the expression levels measured by RNAseq, the ratio of expression levels between tissues using RNAseq was compared to the ratio of expression as measured by qRT-PCR. For all genes, expression of genes in the hippocampus was measured relative to their expression in the PFC. At first we verified the expression of 11 selected lncRNAs with DE profiles between the hippocampus and PFC based on the *p*-value (adjusted *p*-value < 0.05) from RNAseq analyses (Figure 4B). The hippocampus enriched lncRNA genes were *GM2115* (15 ± 0.2 fold), *GM9968* (8 ± 0.12 fold), *GM13644* (4 ± 0.2 fold), *1500017E21Rik* (3.6 ± 0.1 fold), *F730043M19Rik* (3 ± 0.35 fold), and *2310002F09Rik* (9.7 ± 0.5 fold). Three lncRNAs significantly enriched in the PFC were GM11549 (6 ± 0.1 fold), *A830009L08Rik* (10 ± 0.1 fold), and *GM12371* (6 ± 0.1 fold). There were two more lncRNAs, which were only slightly (~1.5 ± 0.2 fold) but not significantly enriched compared to other lncRNAs described earlier in the PFC (Student's *t*-test; *p* < 0.05). We have compared the fold difference of these 11 lncRNAs obtained from DESeq analysis (Figure S4C) and found that the DE pattern of both qRT-PCR and RNAseq experiments are consistent with each other. Similarly, we studied mRNA genes that are differentially expressed in the hippocampus vs. PFC and found that both qRT-PCR and RNAseq data are in agreement with each other (Figure S4D).

We next analyzed the expression levels of six of these lncRNA in the cerebellum and striatum and compared to the expression levels in the hippocampus and the PFC. We found that most of these selected lncRNAs are differentially expressed between the hippocampus, PFC, cerebellum and striatum [Figure 4C: GM11549 (2 ± 0.1 and 20 ± 0.5), *A830009L08Rik* (6.5 ± 0.3 and 23 ± 0.4), *GM9968* (4.5 ± 0.1 and 4 ± 0.15), *A430018G15Rik* (13 ± 0.25 and 11.5 ± 0.1), *GM14290* (2.5 ± 0.1 and 15 ± 0.7), and *F730043M19Rik* (2.7 ± 0.15 and 1.75 ± 0.1), Student's *t*-test; *p* < 0.05]. In the same way we analyzed the expression fold differences for all six lncRNAs in the cerebellum and striatum, respectively, compared to the PFC [Figure 4D: GM11549 (0.3 ± 0.2 and 3 ± 0.5), *A830009L08Rik* (0.6 ± 0.26 and 2.2 ± 0.35), *GM9968* (33.7 ± 0.1 and 30 ± 0.1), *A430018G15Rik* (7.8 ± 0.1 and 7.2 ± 0.2), *GM14290* (1.7 ± 0.1 and 9.4 ± 0.57), and *F730043M19Rik* (9.8 ± 0.15 and 6 ± 0.12), Student's *t*-test; *p* < 0.05]. These results suggest that different brain regions have characteristic lncRNA expression levels.

### lncRNA expression in the tri-synaptic circuitry of the hippocampus

The results from the above experiments suggested that lncRNAs are differentially expressed between brain regions. We next studied how lncRNAs are differently expressed within a brain region. We first examined the lncRNA expression in dorsal and ventral hippocampus and then in the three specific sub-regions of hippocampus DG (Dentate Gyrus), CA3 (Cornu Ammonis 3), and CA1 (Cornu Ammonis 1)—that constitute the tri-synaptic circuitry. DG, CA3, and CA1 regions contain populations of neurons that are homogeneous and show characteristic electrophysiological properties.

**Figure 5:**
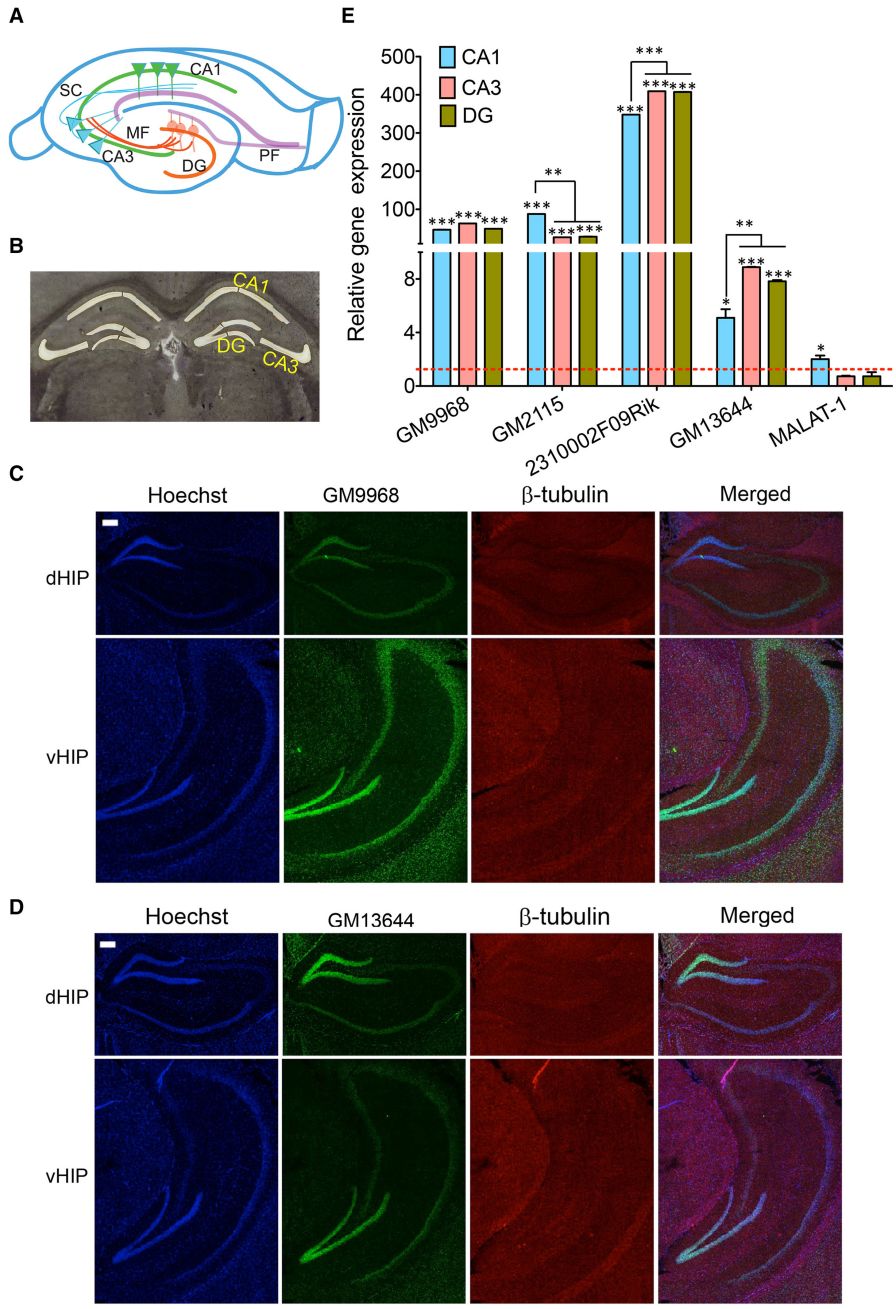
**Subregion specific expression of lncRNAs in the hippocampal tri-synaptic circuitry. (A)** Cartoon of the tri-synaptic circuitry of the hippocampus showing the CA1, CA3, DG sub regions and synaptic connections between DG and CA3 and CA3 and CA1. (DG: dentate gyrus; PF: Perforant path, MF: Mossy fibers and SC: Schaffer collaterals). **(B)** 16 μm coronal sections of the mouse brain, stained with Cresyl violet showing successful laser capture microdissection of CA1, CA3, and DG regions of the mouse brain for qRT-PCR analysis of lncRNA expression. **(C, D)** Are the confocal projection images of coronal section of hippocampus showing expression lncRNAs *GM9968* and *GM13644* in dorsal and ventral hippocampus (dHP: dorsal hippocampus; vHP: ventral hippocampus). Both lncRNAs (both green) co-stained with β-tubulin protein (red), along with the nuclear stain Hoechst (blue) and the merged images are shown. Scale bar, 200 μm. **(E)** qRT-PCR analysis of the five lncRNAs enriched hippocampus in the CA1 CA3, and DG subregions. CA1, CA3, and DG were microdissected **(B)** using LCM. The dotted red line shows 1-fold enrichment. Data was normalized to 18sRNA levels. Bar graphs show enrichment of five lncRNAs (indicated in the Figure) in the three subregions compared to the entire hippocampus (*N* = 4; Student *t*-test, ^*^P-value < 0.05, ^**^P-value < 0.01, ^***^P-value < 0.001). Error bars represent SEM.

Dorsal and ventral hippocampus are implicated in different cognitive functions (Wang et al., 2013). The *in situ* hybridization analyses showed the specific distribution of *GM9968* lncRNA in dorsal and ventral hippocampus. *GM9968* is equally distributed in the DG, CA1, and CA3 regions in the ventral (vHP) as well as dorsal hippocampus (dHP) as seen in Figure 5C. However, between the vHP and dHP, the expression level of GM9968 is different. *GM9968* level appears to be higher in the vHP compared to dHP (Figure 5C). On the other hand, *GM13644*, another lncRNA enriched in the hippocampus, did not exhibit any prevalence in the vHP vs. dHP (Figure 5D). Nonetheless, *GM13644* shows DE patterns between CA1, CA3, and DG in both vHP and dHP. From *in situ* analyses, it is evident that expression of *GM13644* is higher in the DG compared to CA1 and CA3 (Figure 5D).

Next, we isolated the subregions of the hippocampus using laser capture microdissection (LCM) and isolated the RNA for qRT-PCR analysis. A representative Cresyl violet staining image showing successful microdissection of CA1, CA3 and DG regions of the hippocampus is shown Figure 5B. The DE analysis of each region compared to the total hippocampus showed that four of the lncRNAs we analyzed follow a unique expression pattern in a specific sub-region of the hippocampus as summarized in Figure 5C. All of these lncRNAs are enriched in these sub-regions compared to the total hippocampus, which suggests potential roles in spatial regulation of gene expression of lncRNAs. Additionally, we found differences in the expression levels of some of these lncRNAs between CA1, CA3, and DG. For example, *GM2115* is significantly enriched in the CA1 relative to both CA3 and DG (Student's *t*-test, *p* < 0.05) whereas CA3 and DG have similar levels (Figure 5E). In the case of 2*310002F09Rik*, CA1 has significantly lower expression levels (Student's *t*-test, *p* < 0.05) relative to both CA3 and DG (Figure 5E). Another lncRNA, *GM13644*, is significantly enriched in the CA3 and DG compared to CA1 (Student's *t*-test, *p* < 0.05; Figure 5E). These differences in the expression levels indicate neuron specific expression profile of lncRNAs within subregions of the hippocampus. As one of the most abundantly expressed lncRNAs in the hippocampus and PFC, MALAT-1 did not show any preferential expression between these two regions, and within the hippocampal subregions.

qRT-PCR results showed lncRNAs we studied in DG and CA3 have similar expression levels, and expression in CA1 is lower than that in DG and CA3. The slight disparity could come from the challenges posed by LCM during which some amount of RNA degradation unavoidably occurs even under the most stringent RNase-free environment, as well as from the following linear amplification (Wang et al., 2010), where aRNA production leads to shortening of transcripts over successive cycles of amplification (Clement-Ziza et al., 2009). These results indicate a unique spatial distribution of specific lncRNAs within the same anatomical region of the adult mouse brain.

**Figure 6:**
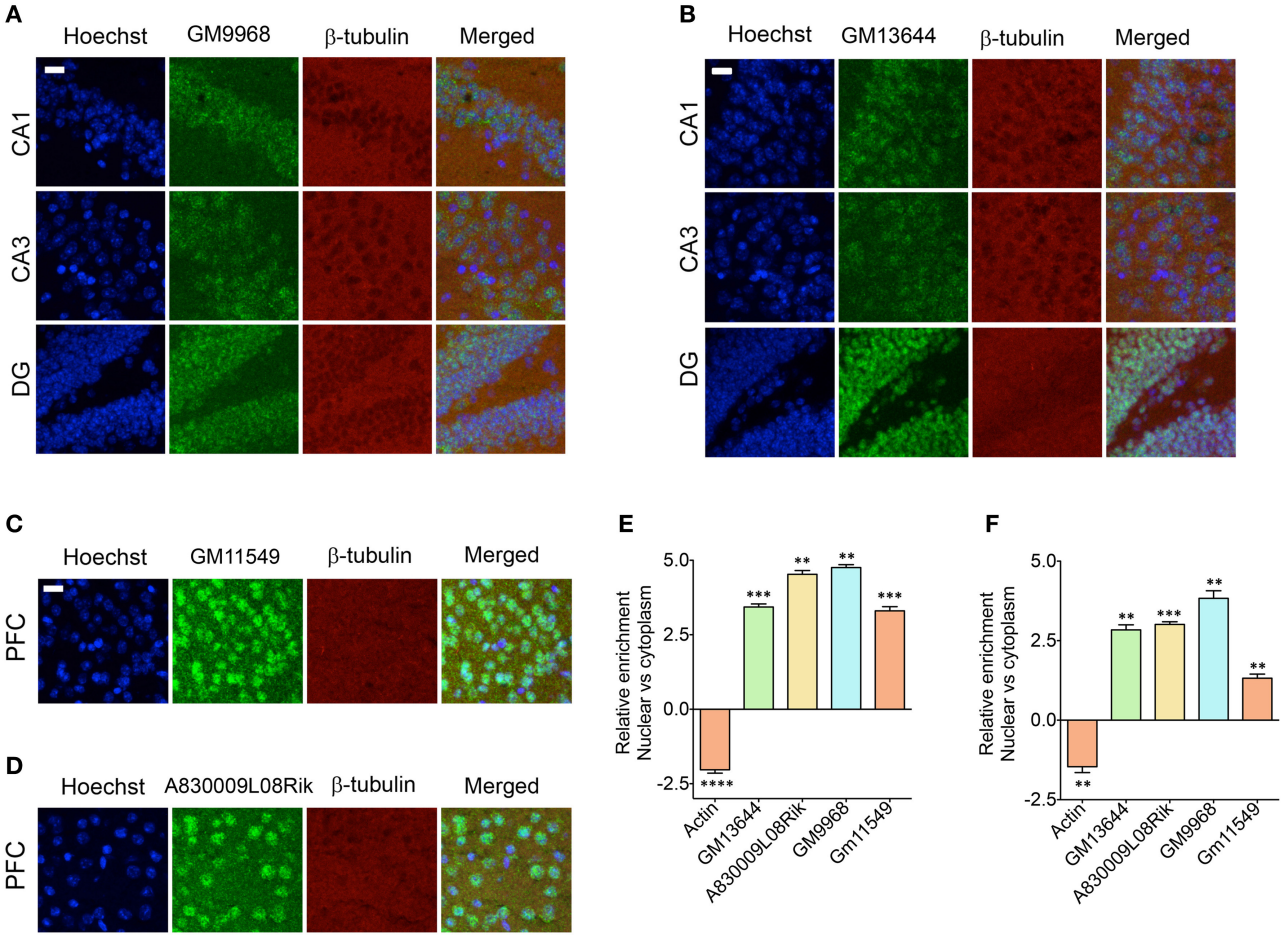
**Subcellular localization of lncRNAs in hippocampus and PFC**. Confocal projection images of coronal section of the hippocampus **(A,B)**, and PFC **(C,D)** show *in situ* hybridization of two lncRNAs (indicated in the Figure) along with immunohistochemical analysis of β-tubulin. The respective lncRNA is in green co-stained with β-tubulin protein (red), along with the nuclear stain Hoechst (blue). Images in **(A,B)** show the subcellular localization of lncRNAs GM9968 and *GM13644* in the CA1, CA3, and DG subregions of the tri-synaptic loop, respectively. **(C,D)** show the subcellular localization of lncRNAs *GM11549* and *A830009L08Rik* in the PFC. Scale bar, 20 μm. **(E,F)** qRT-PCR validation of *GM9968, GM13644, GM11549*, and *A830009L08Rik* transcripts in the nuclear vs. cytoplasmic fractions from hippocampus and PFC. Relative enrichments of lncRNAs were calculated using delta (deltaCT) values where CT values were first normalized to GM11549 to get deltaCT. β-actin was used as a positive control to validate effective fractionation. Error bars are SEM.

It has been shown that the lncRNAs are trafficked to specific subcellular locations (Prasanth et al., 2005; Puthanveettil et al., 2013; Kadakkuzha et al., 2014; Van Heesch et al., 2014), which are directly indicative of their function. We next investigated the subcellular distribution of four different lncRNAs in the CA1, CA3, and DG neurons and CG1 (cingulate cortex 1) region of PFC. For CA1, CA3, and DG neurons, we selected *GM9968* and *GM13644*, which we analyzed earlier for hippocampal subregion specific expression. To analyze the specific subcellular localization in the PFC, we selected two lncRNAs, *GM11549* and *A830009L08Rik* that are enriched in the PFC, relative to the hippocampus (Figure 4A). We found that all four lncRNAs were enriched in the nucleus compared to the cytoplasm (Figures 6A–D). In order to validate the subcellular distribution of lncRNAs observed from *in situ*, we purified RNA from nuclear and cytoplasmic fractions from the hippocampus and PFC followed by qRT-PCR. Figures 6E,F indicate that the qRT-PCR data is in agreement with the *in situ* hybridization analyses and show prevalence of nuclear localization by lncRNAs.

## Discussion

A recent study of a few highly conserved and brain-expressed mouse lncRNA transcripts and their orthologs in birds and marsupial found that unlike protein-coding genes, the sequences, exon structures, and transcriptional start sites for these non-coding genes are all highly variable. Similar large-scale studies have discovered fundamental characteristics of lncRNAs including their low levels of expression, temporal and spatial patterns of expression exons junction modulation, and disease-related splicing events. In a recent RNA-Seq study in Parkinson Disease (PD) patients that sequenced libraries from leukocytes and two brain regions added over 7000 lncRNAs to the human lncRNA database, of which 3495 were co-expressed in the PD brain and leukocytes suggesting the involvement of lncRNA in neurodegenerative diseases (Soreq et al., 2014). Our results and the above mentioned study outcomes are providing framework to find out the diverse mechanisms through which lncRNAs act to regulate protein-coding genes at both the transcriptional and translational levels.

Estimates from the most recent GENCODE project in mouse and humans indicate the presence of 4074 and 13,870 lncRNAs, respectively. Unlike the numbers of protein coding genes, which do not increase with organism complexity, the numbers of lncRNAs increase significantly (Khaitovich et al., 2006). The vast difference in the number of lncRNAs between human and mouse is intriguing as both species have a similar number of protein coding genes, and implies the role of lncRNAs in evolutionary development. Confirming this notion, it has been reported that that changes in lncRNA sequences constitute half of all the genetic differences between the human and chimpanzee genomes (10). However, it has yet to be determined if these changes are causative of the differences between humans and chimpanzees or if they are the result of genetic drift (Carninci et al., 2005). Despite their significance little is known about the functions and identity of lncRNAs in specific brain regions and specific neuronal populations of the vertebrate brain. Specifically, we do not have unbiased estimates of populations of lncRNAs in sub-regions of the vertebrate brain. To address this, we have carried out RNAseq analysis of the total transcriptome to identify lncRNA population in two key regions of the brain, hippocampus, and PFC of the adult mouse. Annotation of lncRNAs in the mouse genome is yet to be completed and is often challenging due to the low expression levels of these transcripts, which leads to poor definition of the transcript boundaries.

To compare the overall expression status of lncRNAs and mRNAs in the hippocampus and PFC, we employed whole transcriptome sequencing and identified a total of 20,618 genes in the hippocampus and 20,025 in the PFC, including 2759 lncRNAs in the hippocampus and 2561 lncRNAs in the PFC. Less restrictive values for compared methodologies usually results in far too large a number of selected genes and here we applied a rather conservative adjusted *P*-value of 0.05 instead of *P*-value to identify the lncRNAs in these two regions. These lncRNA transcripts reported from the present study, out of the 3823 annotated lncRNA transcripts in the mouse genome, according to NCBIM37, represent a very robust (~70%) expression pattern of lncRNAs in the adult mouse brain. Consistent with the previous results that lncRNAs are generally expressed at lower levels than protein-coding genes and are more likely to display tissue-specific pattern of expression (Mercer et al., 2008; Derrien et al., 2012; Kutter et al., 2012), based on FPKM values we find that more than 80% of lncRNAs are expressed much lower than mRNAs (Figure S1) present in the same brain regions. Our depth of sequencing restricted us from identifying novel lncRNAs and recently described circular non-coding RNAs such as competing endogenous RNAs (Jeck et al., 2013).

Consistent with previous reports (Cabili et al., 2011; Derrien et al., 2012), we found that most of the lncRNA expressed (53%) belong to the lincRNA category followed by antisense RNAs and a low prevalence of other biotypes in both the hippocampus and PFC. We next mapped all the lncRNAs from the hippocampus and PFC onto mouse chromosomes and found that they are present as clusters in varying numbers along the chromosomes, with three chromosomes (Figure S3: 2, 4, 11) expressing >300 lncRNAs and the rest of the chromosomes contained loci for 50–150 lncRNAs. These results are consistent with previous finding on mRNAs and lncRNA pairs in the developing mouse brain that a large number of brain-expressed lncRNA loci are not evenly distributed along the mouse chromosomes (Ponjavic et al., 2009).

**Figure 7:**
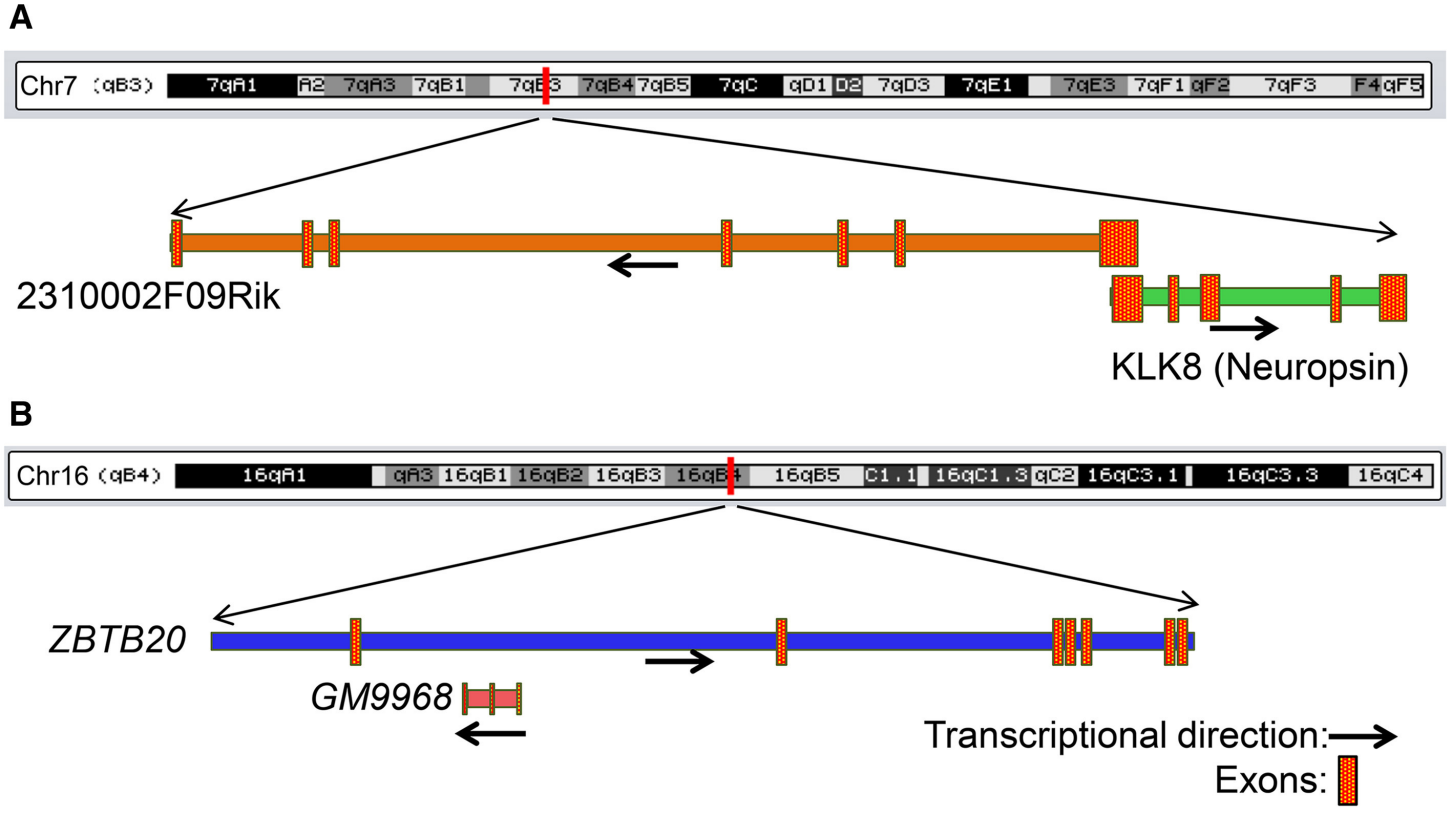
**Genomic context of two lncRNAs that share genomic loci with protein-coding genes associated with neurological diseases. (A)** Genomic locus of the mRNA *KLK8* (neuropsin) and lncRNA *2310002F09Rik* on chromosome 7. *KLK8*-*2310002F09Rik* pair has a 5′ exon overlap and both genes are enriched in the hippocampus. KLK8 is implicated in Epilepsy and Alzheimer disease. **(B)** Genomic locus of the mRNA *ZBTB20* and lncRNA *GM9968* on chromosome 16. *GM9968* completely overlap with *ZBTB20* in antisense direction. *ZBTB20* is implicated in major depressive disorder. The arrow represents the direction of transcription and the boxes represent exons.

We next searched for mRNAs and miRNAs within the 50 Kb of loci of differentially enriched lncRNAs to examine possible co-regulation of expression. We identified three lncRNA–mRNA pairs [*GM9968 and ZBTB20;* 2*310002F09Rik and KLK8* (neuropsin); *GM14290 and KCNB1*] that displayed concordant expression. The observation that these three pairs of tightly co-expressed, share common genomic loci, and are differentially expressed between the hippocampus and PFC, suggest that these lncRNAs might regulate neuronal functions mediated by these mRNAs. For example, the lncRNA *2310002F09Rik*, which is enriched in the hippocampus and the protein-coding gene KLK8/neuropsin, share a 5′ exonic overlap in the antisense direction (Figure 7A). Neuropsin is key in hippocampal plasticity (Oka et al., 2002) and plays an important role in brain diseases, such as Alzheimer's disease and Epilepsy. Another example is *GM9968*, an lncRNA that is significantly enriched in the mouse hippocampus. *GM9968* completely overlaps with *ZBTB20* in the antisense direction (Figure 7B). Previous studies showed that *ZBTB20* in mature CA1 plays an important role in LTP and memory by regulating *NMDAR* activity, and activating *ERK* and *CREB* in mice (Ren et al., 2012). In addition, hypermethylation in the *ZBTB20* gene is shown to be associated with major depressive disorders. Taken together, these observations suggest that by regulating expression of specific mRNAs these lncRNAs might engage in neural plasticity and neuropsychiatric disorders.

Focusing on a subset of lncRNAs, we find that different brain regions and neuronal populations have characteristic lncRNA expression profiles. We examined expression differences in striatum, cerebellum, PFC, hippocampus and differences between dorsal and vHP and within the well-studied tri-synaptic circuitry of the hippocampus. The mammalian brain harbors a large variety of neuron types and sub-types, arranged in specific layers within the sub-regions. The hippocampus is an intensively studied part of the brain with dedicated sub-regions that differ in their physiological functions and capacities and display quite a specialized response to pathophysiological stimuli as illustrated in neurodegenerative disorders. Our studies showed an interesting distribution of these lncRNAs in the tri-synaptic circuitry. The two hippocampus enriched lncRNAs, *GM9968* and *GM13644*, showed different expression patterns. *GM9968* is equally distributed in the DG, CA1, and CA3 regions, but enriched in the vHP compared to the dHP. *GM13644* on the other hand, is equally distributed in the vHP and dHP, but highly enriched in DG compared to CA1 and CA3. Thus, lncRNAs show DE along dorsal-ventral axis of the hippocampus (Figure 5). Consistent with these observations we find that specific neuronal populations in hippocampus have characteristic lncRNA expression profiles. Our *in situ* hybridization and sub-cellular fraction analysis add another layer to the complexity of lncRNA expression. These studies suggest that lncRNAs could be enriched in specific cellular compartments. Taken together, the tissue-specific expression patterns and the distinct subcellular localization of many lncRNAs suggest that their expression is under precise control. We now provide novel details on the expression patterns of lncRNAs per hippocampal sub-region and are of potential interest in further investigation of how lncRNAs regulate gene expression and other hippocampus dependent functions such as learning and memory.

In summary, in this study, we provide the first unbiased estimates of lncRNAs in two key brain regions, the hippocampus and PFC that are involved in memory and cognition. We have characterized their expression, in detail, including their chromosome distribution, genomic context, sub-region specific analyses and subcellular distribution. Our analyses establish complex lncRNA expression profiles in the mouse brain. This DE might play important roles in the development, physiology, plasticity and pathology of the mammalian brain. These findings provide a foundation for further research into the molecular mechanisms of lncRNAs in mammalian brain functions.

## Materials and methods

### Animals

7–9 week old male C57Bl/J6 mice (Jackson Laboratories) were housed in groups of four on a 12 hr. Light/dark cycle with *ad libitum* access to food and water. All experiments were performed during the light part of the diurnal cycle. Housing, animal care and experimental procedures were consistent with the Guide for the Care and Use of Laboratory Animals and approved by the Institutional Animal Care and Use Committee of the Scripps Research Institute.

### RNA preparation for next generation sequencing (NGS)

A total of 8 mice were sacrificed and the respective brain tissues from two mice each were pooled together, providing the sample size of 4 for NGS. 8–12 week old male C57Bl/J6 mice (Jackson Laboratories) were sacrificed and the brain was removed from the skull and placed in ice-cold HEPES HBSS (HHBSS: 1× Hank's basal salt solution, 2.5 mm HEPES-KOH, pH 7.4, 35 mm glucose, and 4 mm NaHCO3). The hippocampus and PFC was dissected and homogenized in Trizol using a glass-Teflon homogenizer containing 1 ml of ice-cold Trizol (Life technologies, USA). RNA was extracted from each fraction using the Trizol-Chloroform method (Life technologies) and stored at −80°C until used.

### Next generation sequencing and data analysis

Beginning with a 200 ng total RNA sample, following the removal of ribosomal RNAs, the sample was reverse transcribed with random hexamer and poly A primers. This product was converted into double stranded cDNA, followed by SPIA amplification. After purification the sample was end repaired and then had specific adapters ligated to the 5′ and 3′ ends. These products are then PCR amplified to enrich for the ligated products (Nugen San Diego, CA). The amplified samples were then purified and diluted down to a 10 μM library. This library was then treated with NaOH to produce single stranded amplicons, which were then added to the Illumina cBOT instrument to generate the double stranded clusters used for the massively parallel sequencing on the Illumina Genome Analyzer IIx according to manufacturer specifications. The read length used for RNA seq is 75 bp.

Analysis of the RNAseq data was performed using Tophat2 (v.2.0.9) (Kim et al., 2013) and the Cufflinks suite (v.2.1.1) (Trapnell et al., 2010). Trimming of the data was performed using the FastX-Toolkit (v.0.0.13) (Toolkit^1^ by Hannon Lab.). The reads were trimmed using the quality scores (cut-off Phred score = 28). The trimmed reads were aligned to the Mus musculus genome build NCBIM37 using Tophat. Tophat was run using its default parameters, which include a maximum of 2 mismatches, a maximum gap length of 2 gaps and an edit distance of 2 for the final alignment.

After the alignment, Cufflinks was utilized to calculate the FPKM values. The default parameters were also used to run cufflinks, except for one parameter “–max-bundle-frags” that was changed to 100,000,000. Cufflinks measure transcript abundances in Fragment Per Kilobases of transcripts per million fragments mapped. The alignment data were then processed and quantified using HTSeq (Anders et al., 2015). The raw read counts generated in HTSeq were used to identify differentially expressed genes in the R Bioconductor package DESeq (Anders and Huber, 2010). HTSeq count was run with “–stranded = no” option, and the ID attribute option, “-i” set to “gene_name.” DESeq uses the total size of each library to normalize the raw read counts and perform calculations on fold change as well as significance based on *p*-value and adjusted *p*-value. The comparisons in this case are between samples of two groups, hippocampus and PFC. The distance matrix analysis based on DESeq method indicates that the hippocampus samples and PFC samples cluster together (Figure S2). LncRNA transcripts were extracted from the Ensembl annotation file (NCBIM37) and categorized into eight biotypes.

### Co-expression analysis/IPA/chromosome distribution

Twenty-four differentially expressed long non-coding RNAs (adjusted *p*-value < 0.05) were selected for co-expression analysis. At first, all the protein coding genes flanking each lncRNA within 50 Kb chromosomal region was selected from the UCSC genome browser. A total of nine neighboring differentially expressed protein-coding genes within 50 Kb distance from these lncRNAs were identified using the UCSC genome browser. To generate heat maps of these two corresponding gene lists (Figures 2A,B), the GeneSpring GX V12.1 (Agilent Technologies) two dimensional hierarchical clustering algorithm was applied to median centered DESeq log2 normalized read counts (yellow/black/blue indicate higher, median and lower expression, respectively). The similarity measure was set to Pearson centered and the linkage rule was set to average.

Ingenuity Pathway Analysis software (Qiagen-IPA) was used to identify enriched canonical pathways in the list of up-regulated (log2 fold change >1, adjusted *p*-value < 0.05) and down-regulated genes (log2 fold change <−1, adjusted *p*-value < 0.05) in the hippocampus compared to PFC. The top 10 up-regulated and down-regulated pathways were identified and sorted according to their statistical significance as the negative logarithm of *p*-values calculated by IPA.

A karyotype displaying the location of all lncRNA transcripts was prepared using IGV (Integrated Genomics Viewer) (Robinson et al., 2011). The loci of lncRNAs were obtained from cufflinks and this information was used to create a BED file. This BED file, along with the karyotype from reference genome mm9, was used to create the karyotype of lncRNA transcripts.

### Quantitative real-time PCR (qRT-PCR)

RNA was isolated from the tissues dissected from the adult mouse brain as described earlier and cDNA was generated by reverse transcription as described earlier (Kadakkuzha et al., 2013). Briefly, 1 μg of RNA was used with Quanta cDNA SuperMix (Quanta Biosciences, Gaithersburg, MD) according to the manufacturer's instructions and the expression of transcripts were quantified by qRT-PCR using SYBR Green PCR master mix (Applied Biosystems Carlsbad, CA) for detection in ABI 7900 cycler (Applied Biosystems Carlsbad, CA). All the qPCR amplifications were performed in quadruplicate in a total volume of 10 μl containing 2 μl of H2O, 2 μl of cDNA, 5 μl of 2X Master Mix, 1.0 μl of 10 μM (each) forward and reverse primers. Quantification of each transcript was normalized to the mouse 18S reference gene following the 2^−ΔΔCt^ method (Livak and Schmittgen, 2001). Student *t*-test was used to select genes with statistically significant expression levels where ^*^p-value < 0.05, ^**^p-value < 0.01, ^***^p-value < 0.001. The sequences of primers for the mRNAs and lncRNAs are given in the Table S3.

### Dissection and freezing of mouse brains for *in situ* hybridization

7–9 week old C57BL/6J male mice were used here. Mice were sacrificed and the brain was removed from the skull and quickly washed in ice-cold HEPES HBSS (HHBSS: 1× Hank's basal salt solution, 2.5 mm HEPES-KOH, pH 7.4, 35 mm glucose, and 4 mm NaHCO3) containing 100 μg/ml of cycloheximide, protease inhibitors (1 tablet/50 ml, complete EDTA-free from Roche Products, Hertfordshire, UK) and SUPER•aseIn™ RNase Inhibitor (1U/μl), Life Technologies, Grand Island, NY). Then, brains were embedded in OCT FSC 22 Blue frozen section compound (Leica Biosystems, Richmond, IL, USA) and immediately frozen at −80°C.

### Cryosectioning

The fresh, frozen brains were sectioned at 16 μm on a Leica 3050 s cryostat. This thickness was optimal for minimizing sectioning artifacts, and was adequate for probe penetration into the section during the *in situ* hybridization procedure. Each OCT block containing a fresh, frozen brain was trimmed in the cryostat until reaching the desired starting section. Two brains sectioned in coronal plane were used to generate 3 series of 24 slides each. Each series contained sections collected from the same brain area (dHP, vHP, and PFC) and each slide of a given series contained four 16 μm thick sections (2 slices per brain).

### *In situ* hybridization

DIG labeled ribo probes complementary to selected lncRNAs for *in situ* hybridization were prepared by *in vitro* transcription of cDNA templates by using T7 RNA polymerase as described earlier (Kadakkuzha et al., 2014). Briefly ~400 nt long sense strand of each transcript was prepared by PCR using mouse hippocampus cDNA as a template and transcript specific PCR primers (Table S4) and ligated to pCRII-TOPO Vector with dual promoters T7 and SP6 (Invitrogen, Cat. Number K4600-01). The Vector with the sense lncRNA DNA in the 5–3′ direction was linearized with BamH I (New England Biolab) for transcription using T7 RNA polymerase to generate antisense probes. A small aliquot (2 μl) was run on 1.5% agarose gel to confirm the integrity of RNA probes and stored at −80°C until used. The *in situ* hybridization using digoxigenin (DIG) labeled riboprobes was followed as described in Puthanveettil et al. (2013). Fifteen μM Cryo-sections were fixed in a freshly prepared solution of 4% paraformaldehyde for 15 min at room temperature, washed three times for 5 min in 0.01 M PBS_DEPC_ and acetylated in freshly prepared TEA solution for 10 min and pre-hybridization at 68°C overnight. After hybridization, the antisense RNA probes were visualized using a Fluorescent Antibody Enhancer kit (Roche, Basel, Switzerland) for DIG detection. The immunohistochemical analyses of b-tubulin were performed for each slice. Alexa Fluor® 546 Donkey Anti-Mouse IgG secondary antibodies (Life Technologies) were used for visualization. Images were acquired by using Zeiss LSM 780 confocal microscope system with 20X/63X objective. Only projection images are shown.

### Laser capture microdissection (LCM)

7–9 week old C57BL/6J male mice brains were freshly frozen in OCT compound and sectioned at 14 μM on Leica 3050 s cryostat, taking every other section throughout the hippocampus. Sections were then mounted on PEN membrane slides, ~12 sections per slide with 3 slides per brain. The staining and preparation of sections for the LCM procedure was done using the LCM staining kit (Cat# AM1935, Life Technologies) with Cresyl Violet following manufacturer's recommendations. Following staining, slides were kept at room temperature before beginning LCM. The microdissection of the CA1, CA3 and DG hippocampal regions were done using the Arcturus PixCell II LCM microscope where the laser power was set at 50 mW and each capture was done with 2 or fewer laser pulses. After dissection, each sample was placed in 50 μl ice-cold Trizol and RNA was extracted using the Trizol-Chloroform method (Life technologies). Purified RNA from each dissection was subjected to a single round of linear T7 RNA Polymerase–driven transcription using MessageAmp™ II aRNA Amplification Kit (Cat# AM1751, Life Technologies).

### Cytoplasmic and nuclear separation of lncRNAs from PFC and hippocampus

PFC and hippocampi of two adult mice were used to extract the total RNA. The tissues were dissected from the whole brain and briefly washed in 1X PBS and immediately transferred into a mortar containing liquid nitrogen to cover the sample and to thoroughly grind the tissues. After the grinding was complete, the liquid nitrogen was allowed to evaporate off without allowing the tissues to thaw. Cell lysis and extraction of cytoplasmic and Nuclear RNA were carried out using SurePrep, Nuclear or Cytoplasmic RNA Purification Kit (Cat. # BP2805-25, Fisher Bioreagents) according to the kit protocol (Jiang et al., 2014). cDNAs were generated from the purified RNA by reverse transcription using Quanta cDNA SuperMix as described earlier and the expression levels of each gene were determined by qPCR using SYBR Green PCR master mix as described earlier. CT values of each lncRNA were first normalized to GM11549, resulting in a deltaCT value. Relative differences between lncRNAs and GM11549 were determined by subtracting the delta CTs of cytoplasm fraction from the delta CTs of nucleus fraction for each sample, resulting in delta (deltaCT) values (Van Heesch et al., 2014). β-actin mRNA that is enriched cytoplasm was used as a control to validate the efficacy of nuclear and cytoplasmic fractionation.

## Author contributions

By performed RNA sequencing; MF, GS, and ACC performed the RNAseq data analyses; BMK, XL, VR, BR, and SVP designed the experiments. BMK, XL, AA, JM, VR, and BR conducted the experiments. SVP designed and directed the project. BMK and SVP wrote the manuscript with input from all the authors. All authors read and approved the final manuscript.

### Conflict of interest statement

The authors declare that the research was conducted in the absence of any commercial or financial relationships that could be construed as a potential conflict of interest.

## Footnotes

^1^http://hannonlab.cshl.edu/fastx_toolkit/index.html

## Supplementary material

### Data availability

RNAseq data reported here is deposited in the GEO database with the accession number GSE61401.

## Abbreviations

DE: Differential expression
FPKM: fragments per kilobase of transcript per million fragments sequenced
GEO: Gene Expression Omnibus
LCM: Laser capture microdissection
LncRNA: Long non-coding RNA
LincRNA: long intergenic non-coding RNA
ncRNA: Non-coding RNA
PFC: Pre-frontal cortex
qRT-PCR: Quantitative real-time polymerase chain reaction.
