# Supplementary material for: Transcriptome analyses of adult mouse brain reveal enrichment of lncRNAs in specific brain regions and neuronal populations
Source: Front Cell Neurosci. 2015 Mar 6;9:63. doi: 10.3389/fncel.2015.00063 (PMC4351618; doi:10.3389/fncel.2015.00063)
Supplement: Supplementary file 5 [file Presentation1.PPTX]

## Slide 1
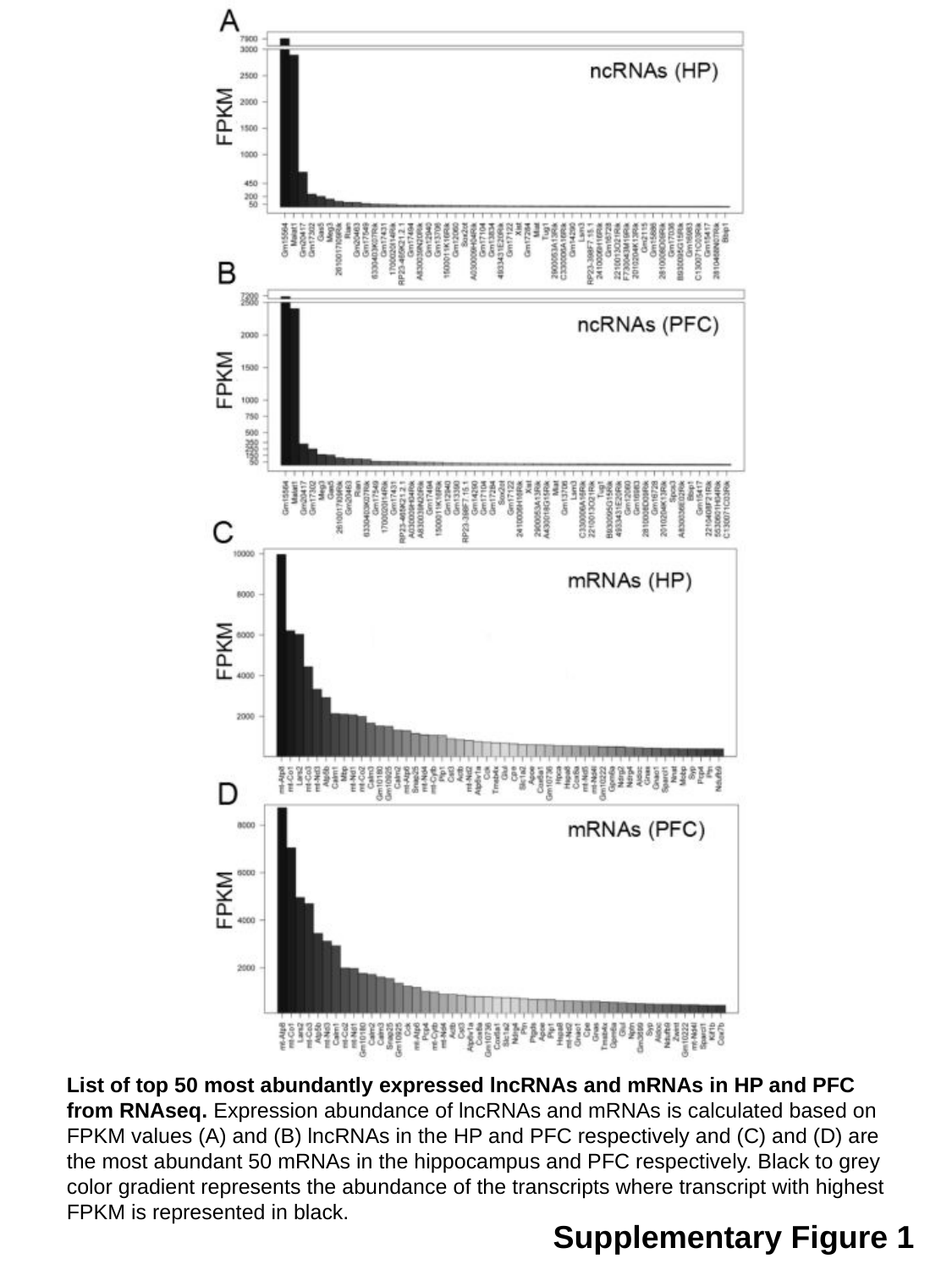

List of top 50 most abundantly expressed lncRNAs and mRNAs in HP and PFC from RNAseq. Expression abundance of lncRNAs and mRNAs is calculated based on FPKM values (A) and (B) lncRNAs in the HP and PFC respectively and (C) and (D) are the most abundant 50 mRNAs in the hippocampus and PFC respectively. Black to grey color gradient represents the abundance of the transcripts where transcript with highest FPKM is represented in black.
Supplementary Figure 1

## Slide 2
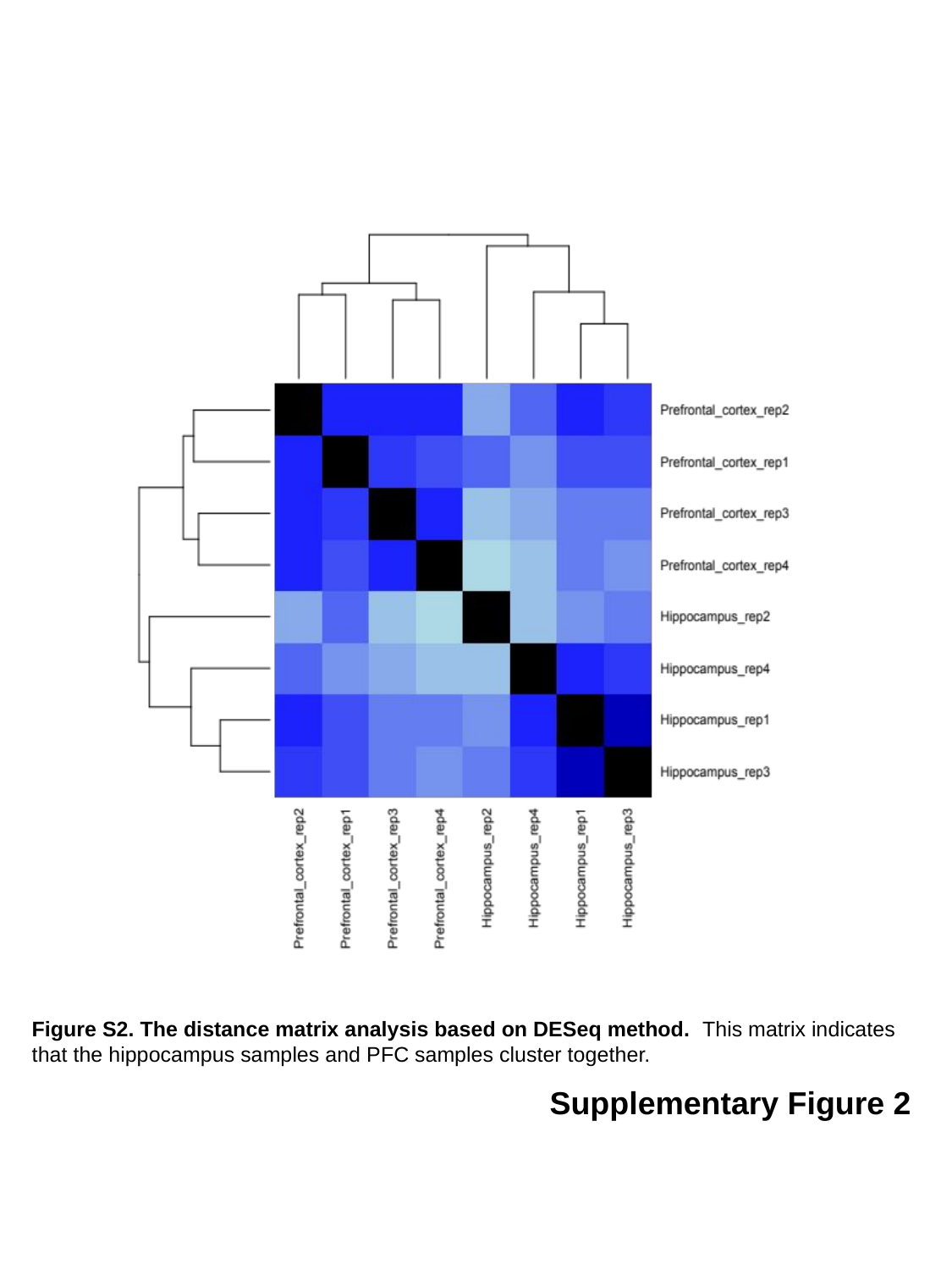

Figure S2. The distance matrix analysis based on DESeq method. This matrix indicates that the hippocampus samples and PFC samples cluster together.
Supplementary Figure 2

## Slide 3
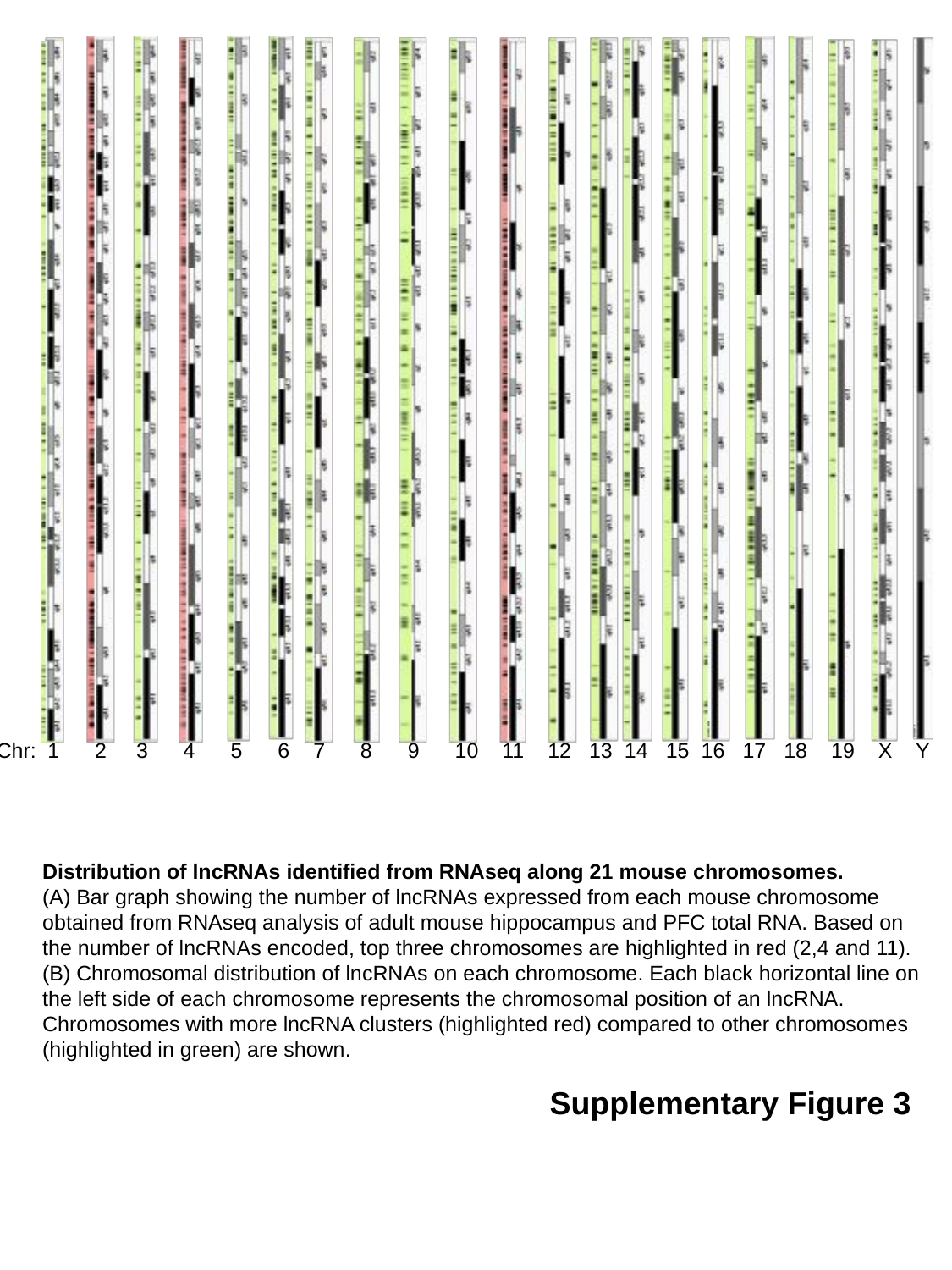

Chr:
 1 2 3 4 5 6 7 8 9 10 11 12 13 14 15 16 17 18 19 X Y
Distribution of lncRNAs identified from RNAseq along 21 mouse chromosomes.
(A) Bar graph showing the number of lncRNAs expressed from each mouse chromosome obtained from RNAseq analysis of adult mouse hippocampus and PFC total RNA. Based on the number of lncRNAs encoded, top three chromosomes are highlighted in red (2,4 and 11). (B) Chromosomal distribution of lncRNAs on each chromosome. Each black horizontal line on the left side of each chromosome represents the chromosomal position of an lncRNA. Chromosomes with more lncRNA clusters (highlighted red) compared to other chromosomes (highlighted in green) are shown.
Supplementary Figure 3

## Slide 4
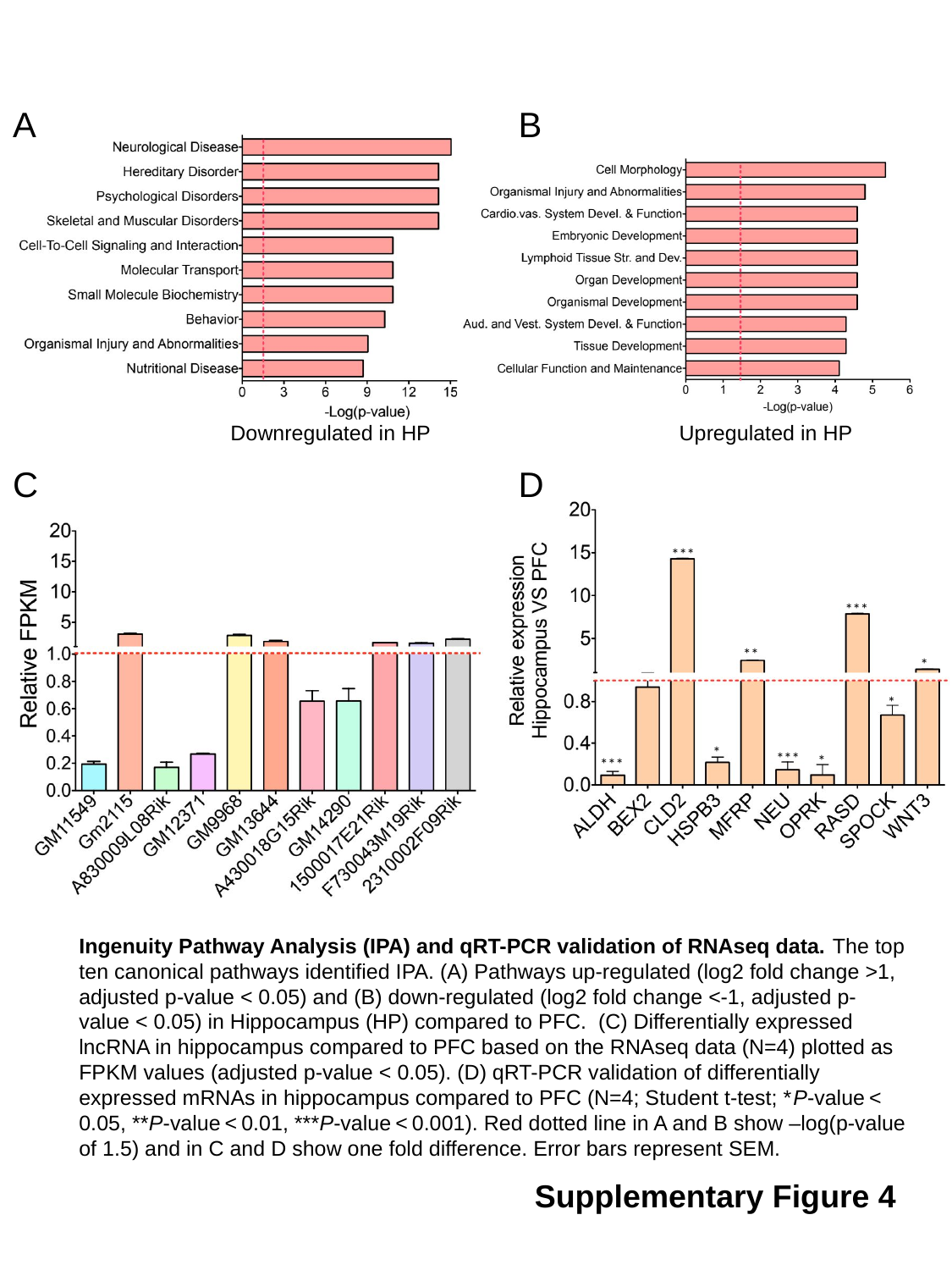

A
B
Upregulated in HP
Downregulated in HP
C
D
***
***
**
*
*
*
***
*
***
Ingenuity Pathway Analysis (IPA) and qRT-PCR validation of RNAseq data. The top ten canonical pathways identified IPA. (A) Pathways up-regulated (log2 fold change >1, adjusted p-value < 0.05) and (B) down-regulated (log2 fold change <-1, adjusted p-value < 0.05) in Hippocampus (HP) compared to PFC. (C) Differentially expressed lncRNA in hippocampus compared to PFC based on the RNAseq data (N=4) plotted as FPKM values (adjusted p-value < 0.05). (D) qRT-PCR validation of differentially expressed mRNAs in hippocampus compared to PFC (N=4; Student t-test; *P-value < 0.05, **P-value < 0.01, ***P-value < 0.001). Red dotted line in A and B show –log(p-value of 1.5) and in C and D show one fold difference. Error bars represent SEM.
Supplementary Figure 4
